# The use of virtual reality in the stimulation of manual function in children with cerebral palsy: a systematic review

**DOI:** 10.1590/1984-0462/2023/41/2021283

**Published:** 2023-03-13

**Authors:** Kharinni Uchôa Pereira, Michelle Zampar Silva, Luzia Iara Pfeifer

**Affiliations:** a Universidade do Estado do Pará, Belém, PA, Brazil.; b Universidade de São Paulo, Ribeirão Preto, SP, Brazil.; c Universidade Federal de São Carlos, São Carlos, SP, Brazil.

**Keywords:** Cerebral palsy, Virtual reality, Hand, Physical functional performance, Daily activities, Paralisia cerebral, Realidade virtual, Mão, Desempenho físico funcional, Atividades diárias

## Abstract

**Objective:**

To identify in national and international literature the use of virtual reality to develop manual skills of children with cerebral palsy.

**Methods:**

This is a systematic review carried out in the PubMed database, Portal de Periódicos da Coordenação de Aperfeiçoamento de Pessoal de Nível Superior (CAPES), and the Online System for Search and Analysis of Medical Literature (Ovid Medline) using the keywords “cerebral palsy”, “virtual reality”, “occupational therapy”, “child”, “daily activities/activities of daily living”, in English and in Portuguese. The selected articles had their methodological quality evaluated through the Physiotherapy Evidence Database (PEDro scale).

**Results:**

228 studies were selected, as they included children with cerebral palsy undergoing treatment with exposure to virtual reality to improve manual function in daily activities. Studies on other themes, incomplete, and duplicated reviews were excluded. Three evaluators conducted the study selection process and included 14 articles in the analysis.

**Conclusions:**

The findings suggest that virtual reality therapy has contributed to an improvement in the manual function of children with cerebral palsy, constituting a useful resource for a supporting intervention to enhance traditional therapies.

## Resumo

**Objetivo:**

Identificar na literatura nacional e internacional o uso da realidade virtual no desenvolvimento de habilidades manuais de crianças com paralisia cerebral.

**Métodos:**

Revisão sistemática realizada nas bases de dados United States National Library of Medicine (PubMed), Portal de Periódicos da Coordenação de Aperfeiçoamento de Pessoal de Nível Superior (CAPES) e no Sistema Online de Busca e Análise de Literatura Médica (Ovid Medline) utilizando as palavras-chave “paralisia cerebral”, “realidade virtual”, “terapia ocupacional”, “criança”, “atividades diárias/ atividades cotidianas”, em inglês e português. A qualidade metodológica dos artigos selecionados foi avaliada pela Physiotherapy Evidence Database (escala PEDro).

**Resultados:**

Foram selecionados 228 estudos que incluíam crianças com paralisia cerebral em tratamento com exposição à realidade virtual para a melhora da função manual nas atividades diárias, sendo excluídos os que estavam fora da temática, os incompletos e os duplicados. Três juízes conduziram o processo de seleção dos trabalhos, incluindo 14 artigos na análise.

**Conclusões:**

Os achados sugerem que a terapia com realidade virtual tem contribuído para a melhora na função manual de crianças com paralisia cerebral, constituindo-se em recurso útil de intervenção coadjuvante potencializadora das terapias tradicionais.

## INTRODUCTION

The International Classification of Functioning, Disability and Health (ICF) considers the human health condition as a dynamic system in which there is an interrelationship between different domains, namely, body functions and structures, activity and participation, and contextual factors (environmental and personal).^1^ Body functions encompass the physiological aspects of systems; body structures encompass the anatomical parts; activity involves action/execution of tasks; participation refers to the individual’s engagement and involvement in life situations; environmental factors involve barriers and facilitators of the physical, social and attitudinal environment; and personal factors are related to individual characteristics.^1^

Cerebral palsy (CP) is the most common physical disability in childhood.^2^ The average prevalence of CP in the United States at the beginning of the century was 3.6 cases per thousand live births;^3^ however, epidemiologists state that in recent years the incidence has decreased by 30% in high-income countries,^4^ reducing the prevalence to 1.4 per thousand live births,^3^ mainly as a result of the combination of comprehensive interventions in obstetric and neonatal intensive care^2^ Although there is reference that seven out of every thousand live births in Brazil have CP^2,5^, these data cannot be confirmed with certainty, since there are very few studies with specific data on prevalence and incidence. The 2010 Census, the last one with available results, establishes that 23.9% of the Brazilian population has some type of disability (totaling 45,606,048 million people), among which 23% have motor disability (impairment of the locomotor system) and 4% have physical disability (complete or partial alteration of one or more segments of the human body)^6^, and CP falls into this classification.

CP triggers motor disorders that may be accompanied by skeletal muscle problems, disorders of perception, cognition, sensations, communication, behavior, and even epilepsy.^7^ These changes in movement and posture^8^ are consequences of changes such as spasticity and muscle weakness and can lead to secondary clinical manifestations such as muscle contractures and shortening.^9^ Thus, all children with CP present with some motor impairment and may have difficulties in tasks that require motor performance.^2^

When compared to their peers, children with CP have around 50% less muscle strength.^10^ Structural muscle changes are observed, including a decrease in the number of sarcomeres in series and muscle volume, with differences in the composition and distribution of muscle fibers that directly influence strength.^7,11^

Among various motor dysfunctions resulting from CP, arm and hand movement difficulties stand out and interfere with these children’s activities of daily living (ADL), as well as school activities.^12^ The lower muscle power output in amplitude more adequate to the function impacts the child’s performance in their activities.^7,10^

The limitations caused by CP motor disorders often interfere with ADL such as self-care, communication, social relationships, transfers, locomotion, recreational and school activities.^7,13^ Some of the limitations of activities are the inability to change postures or body positions, mobility difficulties, inability to move, carry and handle objects with arms, forearms and hands, difficulty in performing basic and independent activities such as eating, personal hygiene and getting dressed.^7,14^ Being interconnected with activities, the participation of children with CP is influenced by their interaction with the environment, whether at home, at school or in the community, and, therefore, is often limited.^13^

Manual function plays a fundamental role in a subject’s interaction with the environment, however children with CP often present abnormal hand postures, such as thumb adduction and/or flexion with limited wrist extension,^15,16^ which in association with increased upper limb tonus can impose significant barriers to activity and participation, impairing functional independence in ADL.^15,17^

There is strong evidence in the literature about intervention strategies to gain effective motor skills in children with CP. Although further studies are still needed, as evidence is classified as weak positive, virtual reality (VR) can increase the positive effects of training when combined with task-specific motor training, as it is motivating, and children report that the interventions with games are pleasurable.^2^

Some studies show that VR used as therapy maximizes hand function of children during ADL,^18,19^ with evidence related to immersion VR for upper limb functional performance variables of quality of movement, selective motor control, postural control, coordination and number of upper limb movements.^19,20^

The use of the tool in VR offers the user with CP the opportunity to experience activities that cannot be performed in the real world, either for safety or for minimizing the limitations inherent to the disability. In this virtual environment, in which it is possible to play without worrying about failures, the child is able to train movements systematically, in an enriched and motivating environment.^21^

Since motivation is a modulator of functional neural plasticity, considered an intrinsic factor in the improvement of motor skills of children with CP,^22^ VR offers the opportunity to control stimuli in the face of real-time feedback with independent practices, enabling prior tests, training, therapeutic interventions, different levels and motivation to the participant exposed to such stimuli, which results in successful performance.^13,23^

Thus, the objective of this study was to identify in the national and international literature the use of VR in the development of manual skills in children with CP.

## METHOD

A systematic review was carried out in the databases that index articles in the health area—PubMed, Portal of Periodicals of the Coordination for the Improvement of Higher Education Personnel (CAPES), Online System of Search and Analysis of Medical Literature (Ovid Medline)—registered on the PROSPERO platform under number CRD42022298580. The articles found in each of the databases listed above were exported to the tool State of the Art through Systematic Review (StArt) version 2.3.4.2, software that helps in the organization of the protocol and of the processes of a systematic review.^24^

The words related to the theme were tested in the databases Health Sciences Descriptors (DeCS) and in Medical Subject Headings (MeSH), so the following descriptors were defined for the search, in Brazilian Portuguese and English: “paralisia cerebral/cerebral palsy”, “terapia ocupacional/occupational therapy”, “realidade virtual/ virtual reality”, “atividades diárias/daily activities”, “atividades cotidianas/activities of daily living”.

The search strategies used involved the association with truncation and boolean operators, as follows: “cerebral palsy” AND “virtual reality” AND “occupational therapy” AND “child”; “cerebral palsy” AND “virtual reality” AND “occupational therapy” AND “daily activities/ activities of daily living” AND child”; the Boolean “NOT” was used with truncation in the descriptor “acidente vascular cerebral”, the search strategy being: “paralisia cerebral” AND “paralisia cerebral” AND “terapia ocupacional” AND “criança” NOT “acidente vascular cerebral”.

Experimental studies related to the theme should met the following criteria for inclusion: approach to techniques or procedures related to the theme of this systematic review and having been published in the five years (2015 to 2020) prior to the selection of studies, carried out in November 2020. Incomplete articles, not found via Virtual Private Network (VPN), published in languages other than English or Portuguese and that did not address the research topic were excluded.

The selection criteria were applied in two steps: the first was based on the specifications of heading and abstract, and the second was the full reading of accepted articles, for analysis and data extraction. Both stages were conducted by two independent evaluators. The articles were included when both evaluators would be in agreement and, in case of disagreement, a third evaluator would define if it should be included or excluded, by consensus.

The Patient, Intervention, Comparison and Outcome method was used (PICOT, where P, or problem/population/patient, stands for CP in childhood; I, or intervention/exposure, is the number of sessions with the use of VR through different consoles; C, or comparison, stands for the studies that used a control group for comparison in a single case comparing themselves or without comparison; O, or outcome, is the analysis of variables by ICF domains, body functions and structures, environmental factors, personal factors, activity and participation, and game analysis; and T, or team, stands for the period of individual intervention of each proposal) to elaborate the research question: “What are the effects of virtual reality on children with cerebral palsy’s manual function performance in ADL?”. The Cochrane Handbook database for systematic review emphasizes the importance of using the method PICOT in bibliographic research, as it helps in the formulation of clinical questioning, making it well-structured and targeted.^25^

The content extracted from the selected articles were: study title, research date, authors’ names, hypotheses, objective, abstract, research method, intervention, tests used, intervention time, time of each session, weekly frequency, games used, console used, population, context, outcome, conclusion, potentialities, limitations, references.

The quality of the selected articles was evaluated using the PEDro scale (Brazilian Portuguese version), which helps to classify the methodological quality of intervention studies (criteria from 2 to 11). Item 1 of the scale is considered as an eligibility criterion only for study participants; items 2 to 11 score 1 according to the option “yes” for compliance with the criterion; therefore, the score oscillates between 0 and 10.^26,27^

## RESULTS

**Figure 1.**
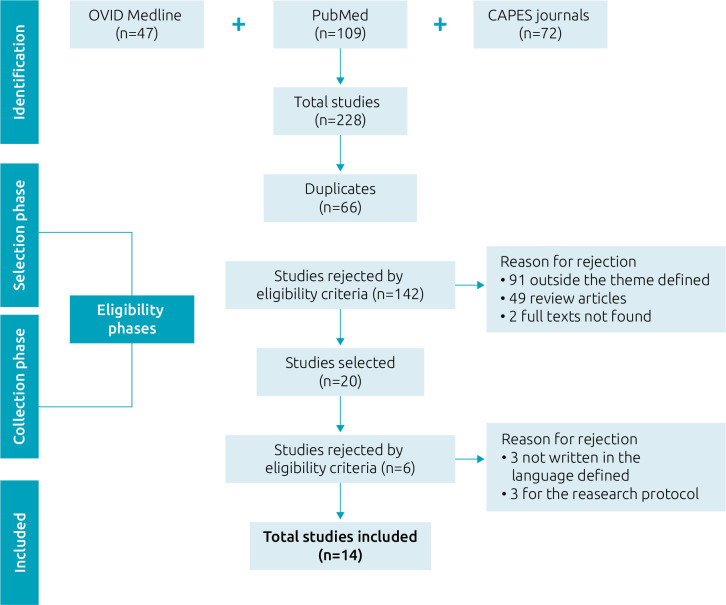
Flowchart of study selection for the analysis.

After searching the databases and analyzing the articles, 14 articles were selected for the review. Figure 1 shows the selection process of the analyzed studies.

OVID Medline: Online Medical Literature Search and Analysis System; CAPES: Coordination for the Improvement of Higher Education Personnel.

Studies from different countries and continents were included, being eight from Europe (Turkey, Italy, Switzerland, Spain, Germany),^28-35^ three from Asia (Jordan, China, Israel),^17,36,37^ two from South America (Brazil)^33,34^ and one from North America (Canada).^38^

Regarding methodologies, five articles were case-control studies;^28,33,36,37,39^ six were single-case experiments;^17,20,29,31,34,35,38^ one study had a cross-sectional design;^40^ another one was a retrospective, single-case, observational cohort study;^32^ and another one was a case-control, unicentric, crossover study.^30^

The methodological quality of the articles, evaluated by the PEDro scale, had an average score of 4.14 points, ranging from 2 to 9. The main factors that reduced the quality score were nine articles being a single study group design, which influenced items related to: hidden and random allocation, participants, therapists and blind evaluators, measurements, and comparison between groups.

The age range for the experimental group found in the research ranged between four^26^ and 19^39^ years of age. The total sample size varied from 8^30,33^ to 60^28^ participants; when considering only the experimental group, a variation between 8^30,33^ and 43^32^ participants was identified.

Participants with CP were classified according to their manual function based on the Manual Ability Classification System (MACS) and their gross motor function by the The Gross Motor Function Classification System – Expanded & Revised (GMFCS – E&R).^41,42^ Four studies^29,32,34,38^ involved children with mild to moderate manual impairment, classified between MACS I and III; two^17,35^ involved children with mild to severe manual impairment, classified between MACS I and IV; and one^39^ was conducted with children with mild manual impairment, classified as MACS I and II. Only three studies reported the gross motor function classification of children and adolescents with mild conditions, classified as MACS I and II;^29,39^ and one study included children with mild and severe conditions, classified between MACS I and IV.^37^ The details of all information presented can be found in Table 1.^17,28-40^

**Table 1.** Identification and methodological quality of studies included.

Authors/year	Sample	PEDro scale
Acar et al.^29^ 2016	CP (30): (6-15 years)	7
Gerber et al.^34^ 2016	CP (13), neuropathy (1); ABI (1): (5-18 years)	2
Kirshner et al.^37^ 2016	CP (15), TD (19): (12 years)	4
van Hedel et al.^35^ 2016	CP (15), Stroke (4), TBI (4), Encephalitis (2), others (9): (5-20 years)	2
Eckert et al.^33^ 2017	EG = CP (1), hypotonia (1), BMD (1), DMD (2), FSH (2), PPS (1): (5-50 years), CG/TD (8): (7-13 years)	3
Hung et al.^17^ 2017	CP (13): (5-15 years)	3
Beretta et al.^31^ 2018	Hemiplegia from severe brain injury (18): (4-8 years)	3
Biffi et al.^32^ 2018	CP (21), ABI (22): (5-18 years)	3
Martins et al.^39^ 2019	EG/CP (10); CG/TD (10): (6-19 years)	4
Şahin et al.^28^ 2020	EG/CP (30); CG/CP (30); (7-16 years)	9
Bortone et al.^30^ 2020	CP or DD (8): (18 years)	8
Daoud et al.^36^ 2020	EG/CP (6): (10 years or +) CG/TD (20): (18 years or +)	4
Leal et. al.^40^ 2020	EG/CP (28); CG/TD (28); (6-15 years)	4
MacIntosh et al.^38^ 2020	CP (19): (8-18 years)	2

CP: cerebral palsy; ABI: ankle-brachial index; TD: typical development; TBI: traumatic brain injury; EG: experimental group; BMD: Becker muscular dystrophy; DMD: Duchenne muscular dystrophy; FSHD: facioscapulohumeral muscular dystrophy; PPS: post-polio syndrome; CG: control group; ABI: acquired brain injury; DD: developmental dyspraxia.

The objectives of the studies selected for review were classified according to the domains of the ICF. Six of them were related to body functions and structures when analyzing the effectiveness of VR in the physiological, affective and emotional aspects;^31-33,35,37,39^ eight were related to environmental and personal factors, as they evaluated the effectiveness of games, consoles, platforms or equipment used in the treatment of certain health conditions such as CP, muscular dystrophy, Becker muscular dystrophy, traumatic brain injury, among other neurodegenerative disorders;^17,28,31,34,36,38^ and, in three studies, the objectives were related to activity and participation, as they analyzed the transfer of skills achieved in virtual games to activities in the real environment.^37,39,40^

The participants’ body functions and structures were evaluated in terms of global motor aspects, manual function and emotional aspects. Motor aspects were measured by the following instruments: Bruininks-Oseretsky Test of Motor Proficiency-Short Form (BOOTMP),^28^ kinesiological assessment,^30^ Bodyfunction^38^ and Gross Motor Function Measure (GMFM).^31^ Manual function was measured by the following instruments: Quality of Upper Extremities Skills Test (QUEST),^17,31,32^ Melbourne Assessment of Unilateral Upper Limb Function,^17,31,32^ Box and Block Test (BBT),^18^ ABILHAND-Kids Test,^17,29^ Jebsen Taylor Hand Function Test in the variable of quality test of skills of upper extremities,^29^ Assessment of the Supporting Hand (AHA)^38^ and Nine Holes Test (9-HPT).^30^ Emotional aspects were measured by the questionnaires: State and Trait Anxiety Inventory (STAI-S), Self-Assessment Manikin (SAM) and Affective Picture System (IAPS).^37^ Activity and participation were assessed by the Canadian Occupational Performance Measure (COPM)^38^ and the Pediatric Functional Independence Measure (self-care) (WeeFIM).^29^

The VR instrument itself was used to quantify results of the intervention in nine studies.^31-37,39,40^ The variables presented were game score, game time, reach of a given task, movement tracking, range of motion, speed and analysis of electromyography by sensors, with greater possibilities for the instrument.

The VR equipment and games used in the studies were quite diverse and are shown in Table 2.^17,28-40^ The period of VR intervention was quite varied as well—from single sessions^33,35,37,39,40^ to a maximum of 12 weeks.^17^ Single sessions ranged from 8 minutes^39^ to 5 hours and 30 minutes,^35^ with frequency from two weekly 60-minute sessions^17^ to a single 90-minute session,^37^ with shorter intervention times of four weeks,^31,38^ but with an increase in weekly frequency to five sessions per week. The duration of procedures is shown in Table 3.^17,28-40^

**Table 2.** Equipment and games used in the studies included.

Authors	Virtual reality equipment	Games
Acar et al.^29^	Nintendo Wii	Tennis, baseball and boxing.
Gerber et al.^34^	YouGrabber® system, portable version	Exergames and Hamster Splash
Kirshner et al.^37^	IREX 2D Camera Tracking VR System	Meal-Maker Virtual Environment
van Hedel et al.^35^	YouGrabber® system	Airplane game
Eckert et al.^33^	Blender Game Engine Platform with Kinect Sensor	The Ladder, The Boat, Whack-a-Mole and The Paper-Bird
Hung et al.^17^	Computer with Scratch software and Kinect2Scratch software + Kinect/Windows Sensor;	Howell; Hungry Shark and Hungry Ant
Beretta et al.^31^	Armeo®Spring	Armeo®Spring (game)
Biffi et al.^32^	Armeo®Spring	Vertical capture
Martins et al.^39^	Computer with *webcam*	Real task: timing. Virtual task: MoveHero
Şahin et al.^28^	Computer + Microsoft Kinect v 1.0t sensor;	Aerial challenge; boxing coach; wall break; Jet run; super kick
Bortone et al.^30^	Equipment VERA	Moneybox and Marble; Labyrinth game
Daoud et al.^36^	Computer + Kinect sensor for Windows v2	Collect stars
Leal et. al.^40^	Computer with touchscreen + Kinect sensor	Verification limit
MacIntosh et al.^38^	ICP	ICP game

ICP: interactive computer play; VERA: immersive virtual environments and wearable haptic devices; VR: virtual reality; IREX: immersive rehabilitation exercise.

**Table 3.** Duration of procedures performed in studies included.

Authors	Intervention period	Session time
Acar et al.^29^	Twice a week for six weeks	45’ (GNW 15’ + routine 30’ ND); and routine GND
Gerber et al.^34^	Two weeks (185 min ± 45 min); Three weeks with varying frequencies	1st meeting with standardized instruction for parents and patient (45’) + home training: 1st week. 5 days/week. for 30’ + 2nd week. with free time.
Kirshner et al.^37^	Single session	90’
van Hedel et al.^35^	Single session	3 steps of 90’’: wide path, narrow 50%, narrow 80% + 30” gap between each step = total 330”
Eckert et al.^33^	Single session OT run the four tested games	Not informed
Hung et al.^17^	Twice a week for 12 weeks	60’: VR (30’) + conventional 30” OT
Beretta et al.^31^	Ft: five times a week for four weeks CIMT: four times a week for four weeks VR: five times a week for four weeks	Ft: 45’ CIMT: at least 3 h/day VR: 45’
Biffi et al.^32^	Ft: 20 sessions for four weeks VR: 20 sessions for four weeks	Ft: 45’ VR: 45’
Martins et al.^39^	Single session	8’
Şahin et al.^28^	Twice a week for eight weeks	45’
Bortone et al.^30^	VR: twice a week for four weeks + rest break of four weeks + traditional therapy twice a week for four weeks	15’
Daoud et al.^36^	180 sessions each group: CP: 12 OT 16 periods (10–15 sessions). TD: three periods (60 sessions)	A game session followed by a 2’ relaxation period
Leal et. al.^40^	Single session	30’ between acquisition phase, rest, retention phase; transfer phase.
MacIntosh et al.^38^	Five times a week for four weeks	1st of 60’; + 3 OT 6 visits (20’–30’)

GNW: Nintendo Wii group; GND: neurodevelopmental group; VR: virtual reality; OT: occupational therapy; Ft: physical therapy; CIMT: constraint-induced movement therapy; CP: cerebral palsy; TD: typical development. ’: minutes; ”: seconds.

## DISCUSSION

In this review, we found evidence of the effectiveness and applicability of VR in children with CP as a tool for stimulation of different skills. Although VR is an effective intervention method to improve manual function (with weak positive evidence) for the treatment of children with CP when associated with task-specific motor training,^2^ in the studies analyzed here, it was proven an important tool to stimulate gains in other domains related to the health condition of children with CP, according to the ICF.^1^

Since ICF considers that the health condition is a product of the interaction between functionality (functions and body structures, activity and participation) and contextual factors (environmental and personal),^1^ the different domains of ICF were identified very clearly among the objectives outlined in the studies analyzed.

The works conducted on this theme are of global interest, since they come from different countries. However, there are few robust studies considered to be of great evidence, as most of them were a single-case experimental study. This was reflected in the low scores of the PEDro quality classification scale, as these scores did not meet all the items considered relevant in this classification. The absence of a comparable “no treatment” control group is a limitation that reduces the power of the results found, making it impossible to establish whether a given intervention is more efficient in promoting functional benefits than other types of training.^17,29^

Among all studies analyzed, only two were randomized controlled trials, five were quasi-experiments, and the rest were single-case or case series experimental studies. Considering the hierarchy of scientific evidence, these studies were classified at levels 2, 5 and 8, respectively.^43^ This is in line with the systematic review by meta-analysis on the evidence of interventions with children with CP, in which it is stated that RV still has weak positive evidence, which suggests the need for more research, which would increase confidence in effect estimation.^2^

As for the domains of ICF, the studies focused on body functions and structures, since most evaluated manual skills (speed, agility, bilateral coordination, strength and reach)^17,29-32,38^ and global motor aspects (coordination, transference, locomotion and balance).^28,30,31,38^ The literature states that VR games are motivators for training upper limb functionality, finger movement and manual function,^44,45^ results that are reinforced in this study. On the other hand, the aspects involved in activity and participation were evaluated in only two cases.^29,38^ The lack of studies focused on this topic is strange, since motor disorders present in CP interfere in the daily activities of self-care, communication, social relationships, recreational and school activities^7,12,13^ and, therefore, should be considered target of interventions.

Many technologies have been used to improve the development of children with CP and implemented by virtual environments, whether immersive, semi-immersive and non-immersive, interactive, motivating and appropriate.^19^ VR programs offer children with CP the possibility of performing activities in a virtual environment that could not be performed in the real world due to limitations or lack of safety.^46^

In the recent past, interventions with VR in rehabilitation involved complex and high-cost systems, which made their application in clinical practice unfeasible, being restricted to research groups. However, with the expansion of video games after the 2000s, they started to be used by researchers as VR equipment in different studies.^47^ The equipment used in the studies of this review were quite diverse; however, many authors have adopted video games and/or computers with attached devices,^10,23,24,28,31,33,34^ that are easily accessible to the general public, which has allowed the incorporation of these resources into clinical and/or in the daily routine of children with CP.

The VR games used in these studies were also very diverse and related to the objective to be achieved with the intervention. For Silva,^47^ virtual games and rehabilitation goals needed to be convergent so that this resource would allow functional gains, since the repetition and variability of movements present in VR games add playfulness to the therapeutic process, increasing motivation and adherence to neurological rehabilitation.

The conclusions of studies regarding the effectiveness of VR in the treatment of several neurological (including CP) and degenerative health conditions can be seen in five articles, in which the therapy with VR had good evidence of efficiency as adjuvant treatment that enhances traditional therapies (occupational therapy and physical therapy).^17,28,29,32,38^ In one study, VR therapy achieved sufficient results to be considered an alternative to traditional therapy.^30^

Four studies proved the efficiency of the equipment and games used^.33,34,36,40^ The studies by Martins et al.^39^ and Beretta et al.^31^ stated that the acquisition of skills in the virtual environment was effective in improving activity and participation. In another article, VR therapy was proven effective both for the equipment and games used and good to be applied as adjunctive therapy to enhance traditional treatment.^37^ In the study by Gerber et al.,^34^ treatment with VR was reported by parents as beneficial, although the authors recognized the need for adjustments due to technical problems with the equipment and the need for games to become more interesting.

Some limitations were pointed out in the studies, such as lack of standardization of evaluation and intervention criteria,^29,34-36,38-40^ equipment technical problems,^17,34^ lack of games adapted to patients with greater limitations,^28,33^ restricted number of games that encourage participation of children and adolescents,^28,33^ small sample size,^30,31,36,37,39^ absence of a control group,^17,29,32^ wide variability (heterogeneous) of group,^32,34^ short-term interventions and lack of a crossover design that would allow a better understanding of the influence of VR on CP therapy.^17,39^

In conclusion, VR is a facilitating device for the development of various motor, emotional and self-care skills in children and adolescents with physical dysfunctions, including CP. It is useful as an adjunct therapeutic resource in traditional therapies, enhancing rehabilitation for the improvement of manual function, as well as improving the involvement in activity and participation in a real environment, autonomy and quality of life. As it is a playful and pleasurable resource, it favors engagement of children and adolescents in therapies, supporting the rehabilitation process. However, the limitations presented in the studies justify further research, especially randomized clinical trials, to assess the effectiveness of VR for greater gains in activity and participation.
